# Positive Murphy’s Sign in Pericardial Hematoma from a Right Atrial Tear

**DOI:** 10.7759/cureus.3402

**Published:** 2018-10-02

**Authors:** Srilekha Sridhara, Christopher Lichtenwalter, Shahnaz Mazdeh, Beeletsega T Yeneneh

**Affiliations:** 1 Internal Medicine, Banner Baywood Medical Center, Mesa, USA; 2 Cardiology, Banner Heart Hospital, Mesa, USA; 3 Internal Medicine, Banner Heart Hospital, Chandler, USA; 4 Cardiology, Maricopa Integrated Health System, Phoenix, USA

**Keywords:** pericardial hematoma, atrial tear, murphy's sign, cardiac failure

## Abstract

A positive Murphy’s sign in a patient with right upper quadrant abdominal pain is the arrest of inspiration during deep palpation of the quadrant. It is usually suggestive of acute cholecystitis. We report an unusual case of a positive Murphy’s sign not due to acute cholecystitis, but rather from a pericardial hematoma from a right atrial tear causing right heart failure. The patient required an atrial tear repair to prevent a cardiac tamponade.

## Introduction

Pericardial hematomas can be secondary to trauma, cardiac surgery, or coronary interventions. Chronic expanding pericardial hematomas are very rare after cardiac surgery and, if not diagnosed in time, can lead to cardiac tamponade and death. We report what we think is the first definitive case of a chronic expanding pericardial hematoma from a right atrial tear and expand on our patient’s clinical presentation and management.

## Case presentation

The patient is a 57-year-old obese female with a history of paroxysmal atrial fibrillation, hypertension, hyperlipidemia, and type 2 diabetes mellitus who was evaluated for symptoms of dyspnea. Cardiac catheterization findings included a chronic total occlusion of the right coronary artery with left to right collateral filling of the distal right coronary artery and significantly obstructive left anterior descending artery stenosis as confirmed by fractional flow reserve. Transesophageal echocardiography (TEE) revealed severe mitral regurgitation which appeared rheumatic in origin. She subsequently underwent mitral valve replacement, two vessel bypass using the left internal mammary artery to left anterior descending artery, and saphenous vein graft to posterior descending artery, left atrial appendage ligation, and left-sided maze procedure. Anticoagulation with coumadin was started on postoperative day 4 for a planned duration of three months. She was discharged on coumadin, aspirin, amiodarone, and metoprolol. She was seen in clinic two and three weeks post-discharge and was doing well with relief of her preoperative symptoms of dyspnea and no evidence of heart failure. However, she returned to the hospital five weeks post-discharge because of the sudden onset of right shoulder pain and right upper quadrant pain. She was noted be in atrial flutter with rapid ventricular response. Given the initial borderline hemodynamic instability, she was electrically cardioverted in the emergency room with a return to sinus rhythm. On this presentation, she was also noted to have hypothermia, leukocytosis, elevated liver enzymes, elevated troponin of 4.80 ng/ml, creatinine 1.57 mg/dl, estimated glomerular filtration rate (eGFR) of 36 mL/min/1.73 m^2^.L, hyponatremia (Na 131 mmol/L), hyperkalemia (K 6.2 mmol/L), and protime/international normalized ratio (PT/INR) 88.9 seconds and 7.2, respectively.

The initial working diagnosis was severe sepsis from possible acute cholecystitis leading to multiorgan damage. An initial workup focused on possible acute cholecystitis as the cause of her symptoms. Ultrasound of the abdomen showed gallbladder wall thickening at 7 mm and positive sonographic Murphy’s sign, with no definite evidence of cholelithiasis. Hepatobiliary scintigraphy showed no evidence of acute cholecystitis with a normal hepatic uptake and excretion of technetium 99 radioisotope, normal gallbladder ejection fraction, and no cystic or common bile duct obstruction. Magnetic resonance cholangiopancreatography showed no evidence of choledocholithiasis or biliary dilation. A computed tomography (CT) scan of the abdomen and pelvis without contrast showed an unremarkable liver, gallbladder, and pancreas but revealed a moderate-sized hemopericardium. Transthoracic echocardiogram (TTE) revealed only a small pericardial effusion. On subsequent days in the hospital, she had worsening dyspnea. Physical exam showed worsening right-sided heart failure. Laboratory evaluation revealed improved leukocytosis and renal function but worsening liver function tests with aspartate aminotransferase level steadily increasing from 27 to 571 to 1,063 and alanine aminotransferase from 22 to 404 to 1,026. It was also difficult to achieve adequate diuresis to alleviate her symptoms.

**Figure 1:**
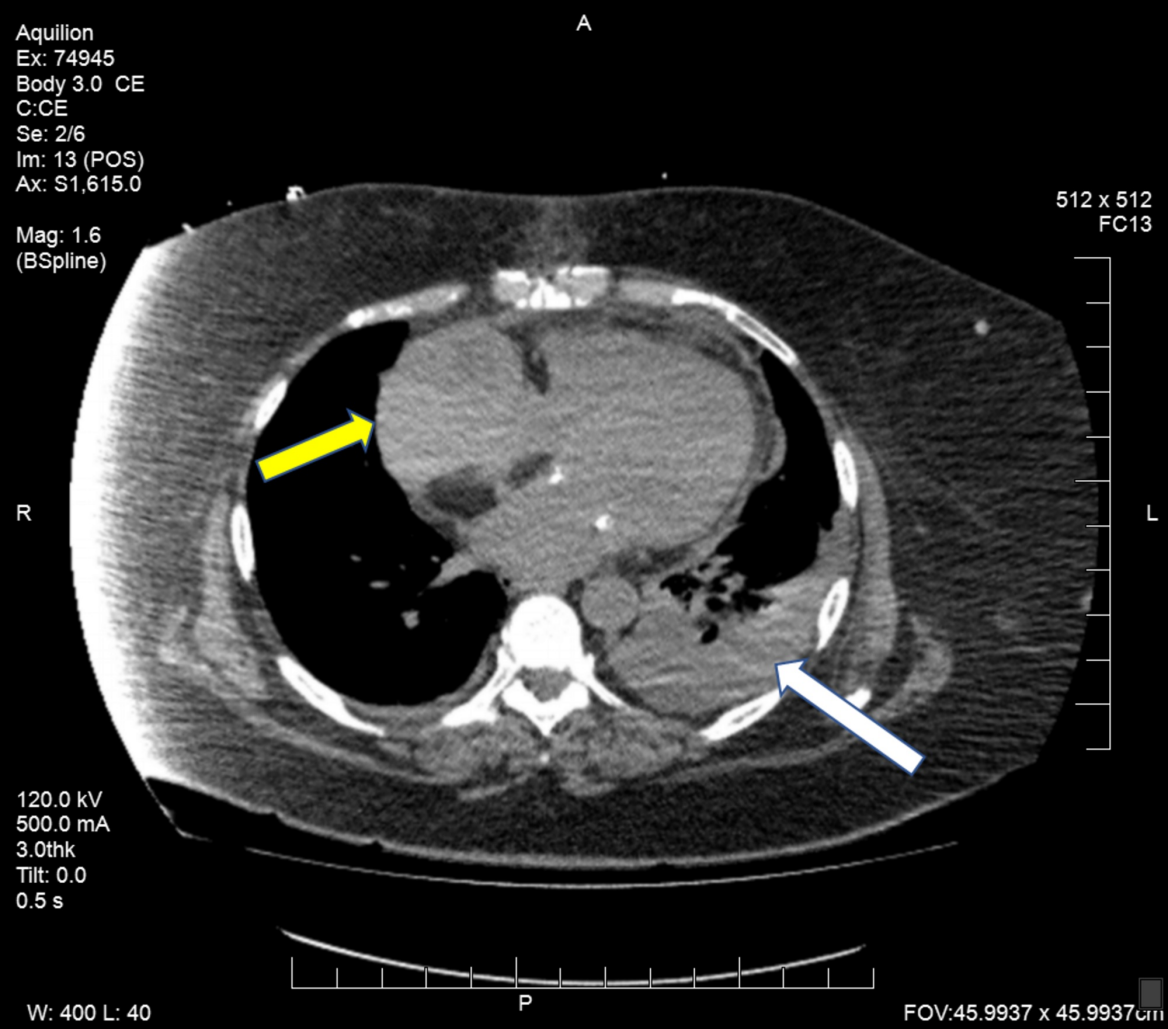
CT scan of the chest showing pericardial hematoma Computed tomography (CT) scan of the chest showing a pericardial hematoma indenting the right atrium (yellow arrow) and left lower lobe pulmonary infiltrate (white arrow)

**Figure 2:**
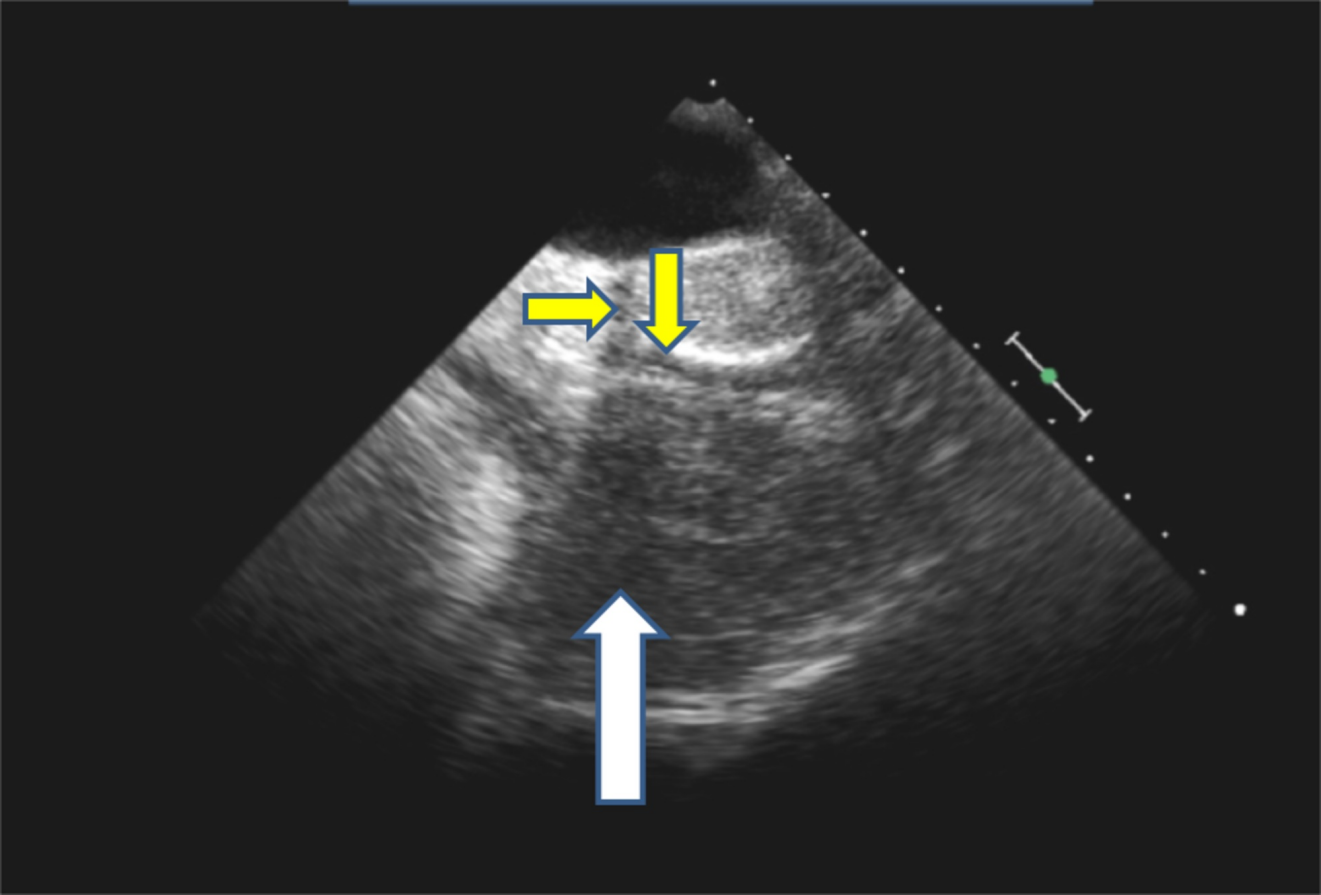
Pre-surgery transesophageal echocardiogram (TEE) image without color flow showing the pericardial hematoma Pre-surgery TEE showing pericardial hematoma (white arrow) compressing and nearly obliterating the right atrium (slit-like shape, yellow arrows). Image seen is without color flow.

**Figure 3:**
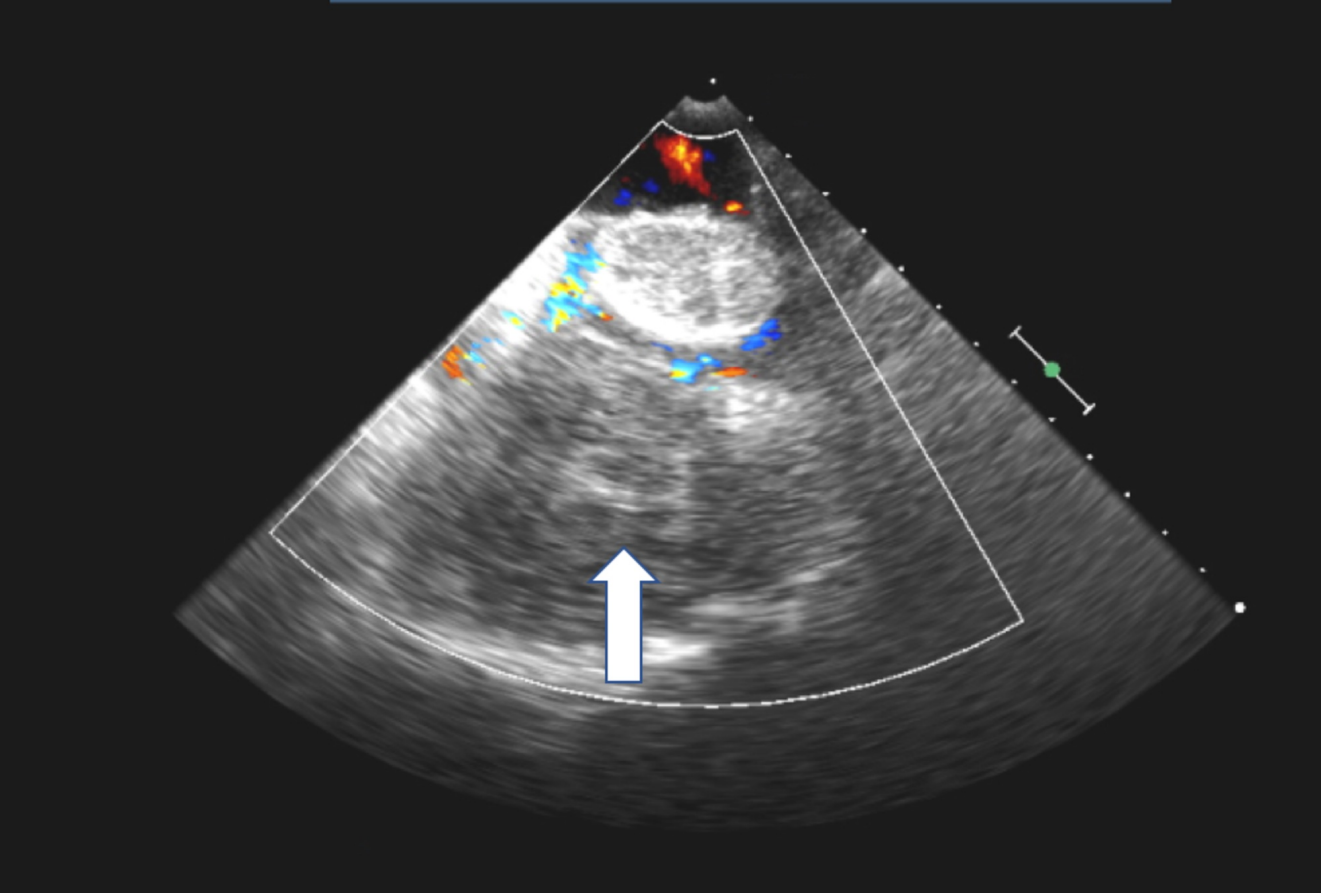
Pre-surgery transesophageal echocardiogram (TEE) image with color flow showing the pericardial hematoma Pre-surgery TEE showing pericardial hematoma (white arrow) compressing and nearly obliterating the right atrium. Image seen is with color flow.

Other explanations were sought for the elevated liver enzymes, and a CT scan of the chest was performed. This showed a 5.1 x 8.26 cm pericardial hematoma indenting the right atrium (Figure 1). Following this, she underwent a TEE which showed a large hematoma compressing the right atrium, causing a gradient on the right atrial inflow of 13 mmHg (Figures 2-3). There was no involvement of the tricuspid valve.

**Figure 4:**
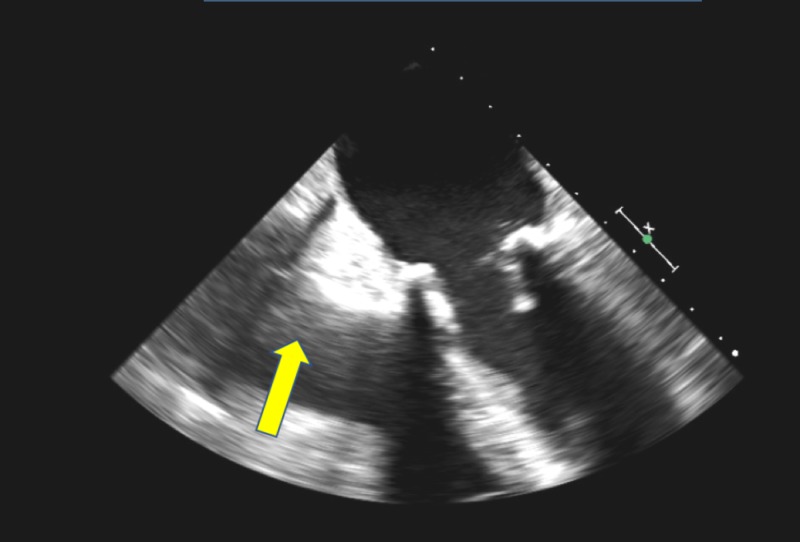
Post-surgery transesophageal echocardiogram (TEE) showing expansion of the right atrium Post-surgery TEE showing expansion and improved filling of the right atrium (yellow arrow) following surgical evacuation of the pericardial hematoma

She then underwent right-sided, video-assisted thoracoscopic surgery (VATS) with exploration and evacuation of the pericardial hematoma. However, given the continued return of bloody fluid from pericardial sac, the VATS was converted to an emergent re-do sternotomy and a 1 cm right atrial tear was located with active bleeding into the pericardium. The right atrial tear was surgically repaired, and the hematoma evacuation was completed. The postoperative course was uneventful. She was easily diuresed over the next couple of days with resolution of the right-sided heart failure. She was discharged home without restarting anticoagulation and has done well over the next several months without any recurrence of pericardial effusion, right heart failure, or atrial fibrillation (Figure 4).

## Discussion

In this discussion, the primary focus is on the delayed development of a pericardial hematoma and cardiac chamber injury following cardiac surgery.

Pericardial effusions resulting in cardiac tamponade after cardiac surgery are uncommon. In a retrospective analysis done by Kuvin et al., 48 (1%) out of 4,561 patients were found to have moderate or large pericardial effusion [1]. Of these, 36 (74%) had evidence of cardiac tamponade. The risk of cardiac tamponade was higher after valve surgery, with or without coronary artery bypass grafting, for the female gender and preoperative use of anticoagulants [1]. Another study noted the incidence of late cardiac tamponade was higher with valve replacement surgery and anticoagulant therapy [2]. A hematoma persisting and increasing in size more than a month after the hemorrhagic incident is considered chronic, and the mechanisms for expanding hematomas seemed to be pericardial injury, irritant effects of blood products, the release of vasoactive substances, capsule formation, and new bleeding from subcapsular microvessels. There are only a few case reports of chronic expanding hematomas after heart surgery. In the report published by Hirai et al., a 63-year-old woman had prior cardiac surgery for a double outlet right ventricle 14 years earlier and for tricuspid regurgitation eight years earlier [3]. She reported cough and chest discomfort, and a CT scan of the chest showed an encapsulated tumor compressing the heart and left lower lung field. She underwent a left thoracotomy and removal of well-circumscribed organized hematoma; the pathology was suggestive of fresh and old hematoma. Her postoperative recovery was uneventful. In the report published by Basha et al., a 61-year-old male presented with dyspnea on exertion, syncope, pedal edema, and decreased urine output [4]. He had undergone cardiac surgery for aortic valve replacement 20 months previously. A TTE showed a 6.54 x 3.12 cm-sized intrapericardial mass posterolateral to the left ventricle, which was compressing on the left ventricle and causing a septal bulge into the right ventricle. He required an anterolateral thoracotomy, partial pericardiectomy, and complete hematoma removal with subsequent expansion of the left ventricle and minimal pericardial collection. Pathology was suggestive of a hematoma with fibrinous material with entrapped red cells, white cells, and areas of cystic degeneration. This patient also had an uneventful recovery.

The majority of pericardial effusions are in the posterior pericardium or near right atrium and cannot be adequately visualized with a TTE, and CT imaging offers better visualization [5]. Kochar et al. reported four postoperative cardiac patients who had developed right atrial compression and were diagnosed using TEE [6]. As in case report by Hirai et al. and our patient, a CT scan of the chest was very important in detecting the pericardial hematoma. Other etiologies of pericardial hematomas, in addition to cardiac surgery, are percutaneous coronary interventions and epicardial ligation device use [7-9].

Coming to cardiac chamber injuries, these can be secondary to blunt trauma [10-12] with cardiopulmonary resuscitation [13] with chest trauma being the most common cause for chamber injury based on our literature search. Delgado et al. reported an atrial tamponade causing an acute ischemic hepatic injury in the immediate postop period after cardiac surgery [14]. To our knowledge, our patient's description is the first case report of a right atrial tear from cardiac surgery presenting six weeks later with a chronic expanding pericardial hematoma leading to hepatic congestion with the presentation mimicking acute cholecystitis with a positive Murphy’s sign. Between the patient’s surgery and her presentation described above, she did not have any cardiac interventions or chest trauma. The atrial tear was probably present, to some level, postoperatively but was clinically silent because it had thrombosed. Coagulopathy noted on her second admission likely caused her to hemorrhage into the pericardial sac, leading to the expanding large pericardial hematoma and causing the extrinsic compression of the right atrium and acute right heart failure. Increased hepatic venous pressure leads to central venous congestion and hepatic sinusoidal congestion. Loss of the atrial kick during the occurrence of the atrial fibrillation further aggravated her condition with some element of hepatic ischemic injury. Although the original presentation appeared to be from sepsis from acalculous cholecystitis, attention to detail regarding the liver enzyme abnormalities in a pattern that was consistent with hepatic ischemic and congestive pattern from worsening right heart failure was essential [15].

## Conclusions

As described above, the risk for post-cardiac surgery pericardial hematoma is higher for the female gender, valve surgery, and anticoagulation therapy. Not locking in on a diagnosis of acute acalculous cholecystitis but looking for alternate causes of transaminitis was important in our patient, especially given the recent extensive cardiac surgery and anticoagulant therapy. Timely intervention with TEE, detection of a pericardial hematoma, and repair of an atrial tear prevented cardiac tamponade, cardiac arrest, and possibly death.
